# The Nexuses Between Social Media Marketing Activities and Consumers’ Engagement Behaviour: A Two-Wave Time-Lagged Study

**DOI:** 10.3389/fpsyg.2022.811282

**Published:** 2022-04-20

**Authors:** Yunfeng Shang, Hina Rehman, Khalid Mehmood, Aidi Xu, Yaser Iftikhar, Yifei Wang, Ridhima Sharma

**Affiliations:** ^1^School of Hospitality, Zhejiang Yuexiu University, Shaoxing, China; ^2^Faculty of Management Sciences, National University of Modern Languages (NUML), Islamabad, Pakistan; ^3^Key Research Base Project of Humanities and Social Sciences of Universities in Hubei Province, Research Center of Hubei Micro and Small Enterprises Development, School of Economics and Management, Hubei Engineering University, Xiaogan, China; ^4^School of International Business, Zhejiang Yuexiu University, Shaoxing, China; ^5^Armed Forces Post Graduate Medical Institute (AFPGMI), National University of Medical Sciences (NUMS), Rawalpindi, Pakistan; ^6^School of Economics and Management, Tongji University, Shanghai, China; ^7^Vivekananda Institute of Professional Studies, New Delhi, India

**Keywords:** perceived social media marketing activities, engagement intention, social media sales intensity, engagement behaviour, stimulus-organism-response (SOR) framework

## Abstract

This study examined how social media marketing activities (SMMA) influence consumers’ engagement behaviour in developing countries. Based on the stimulus-organism-response theory, we examined the effect of SMMA on consumers’ engagement intention and further investigated the moderating effect of social media sales intensity. The study employed a time-lagged design with two waves to confirm the hypothesised framework. The study findings showed that SMMA positively influence consumers’ engagement intention and engagement behaviour. In addition, social media sales intensity strengthens the link between engagement intention and engagement behaviour. This study adds to the literature on social media and discusses its practical implications.

## Introduction

Due to the embedment of technology across the marketing landscape, digital marketing has taken centre stage, resulting in an environment that is very engaging for customers, particularly on social media. Social media marketing is an effective digital marketing strategy that has introduced a new perspective to the current marketing arena. With the explosion of information and communication technology, the face of marketing activities changes as it increases the transformation of product and services in large volumes as compared with traditional marketing practices (Kautish and Rai, 2019). In the past, companies were not sure whether customers viewed or were influenced by their marketing efforts (Kautish and Sharma, 2018). The introduction of social media marketing activities (SMMA) provides a new platform for companies to seek huge target market attention. Social media has progressively become an integral part of our lives in terms of opinion, evaluation, and purchase of any product or service. Companies are moving toward SMMA to approach their specific targeted market with low cost and efficient medium as compared with traditional marketing activities. Globally, 3.78 billion social media users have been noted as of 2021 (Statista, 2021). At present, companies are investing more in these platforms to create a positive image among customers. As COVID-19 impacts the world, most companies are moving toward social media platforms and providing live updates through vlogs, photos, posts, and tweets of new products and services (Vázquez-Martínez et al., 2021). This is because people are confined to their houses due to lockdown and their movement is restricted.

A report published by Hootsuite (2021) stated that there are 4.20 billion active social media users out of the global 7.83 billion population; 81.5% of users searched online for a product or service to buy, 69.4% used a shopping app on a mobile phone, and 76.8% purchased a product online. In Pakistan, the total population is 223 million, of which 46 million are active social media users and 8% of consumers make online purchases. This trend signifies the researcher’s interest in SMMA, preferences, electronic word-of-mouth (eWOM), and purchase decisions (Michaelidou et al., 2011). SMMA are used to make a rational decision; consumers use different SMMA to search for new features and offers generated by companies about the product or services. This is termed as social media information search (SMIS), which refers to the search for information about products or services before making a decision (Xiang and Gretzel, 2010). Social media is becoming a popular source of information (Kim et al., 2013). People use social media to gather information (Westerman et al., 2014). Along with traditional media, social media has been increasingly used for information seeking (Edwards et al., 2014). In this social media era, increased trends in information search, diversified platforms of communication, and dynamic channels of communication make it easy for the company to attract more people (Zhang et al., 2021). SMMA are defined as a “group of Internet-based applications that build on the ideological and technological foundations of Web 2.0, and allows the creation and exchange of user-generated content” (Kaplan and Haenlein, 2010, p. 61). Previously, companies invested a huge budget on marketing campaigns, but as SMMA were introduced, it decreased the cost and reached a large number of geographically dispersed consumers. Companies use SMMA to create awareness about their products and services and share their experiences about the product (Stephen, 2016). SMMA are also used to update consumers of new services or products (Pentina et al., 2018).

Social media has changed the way we interact, share, and communicate with others (Vardeman-Winter and Place, 2015; Gao et al., 2021; Mujahid and Mubarik, 2021; Wang et al., 2021). Social media is “flexible enough to match our social capabilities and new ways of coordinating group action” (Reitz, 2012, p. 43). Social media platforms such as YouTube, Facebook, Instagram, Twitter, and Pinterest have become the most trending platforms for attracting online consumers. This medium provides consumers with the power to share their experiences throughout the world (Kozinets et al., 2010). Companies use SMMA to achieve low-cost marketing objectives (Ajina, 2019). SMMA have become an important source of e-commerce, and they promote relationship building and interactivity among customers (Akman and Mishra, 2017). Social media is used to build and maintain relationships and engage customers in a new way (Toledano and Avidar, 2016). A growing number of companies are using online platforms for customer engagement and connection (Mathwick et al., 2008).

Escobar-Viera et al. (2021) stated that there is limited literature available on social media usage and engagement. An existing study stated that “social media should utilise strategically and take deliberate initiatives to motivate and empower customers to maximise their engagement value and yield superior marketing results” (Li et al., 2021, p. 4). According to the study by Sashi (2012, p. 260), engagement is about “turning on customers by building emotional bonds in relational exchanges with them.” The interactive nature of SMMA establishes a close relationship between companies and customers (Dolan et al., 2019). Customer engagement is a crucial construct in online and social business environments (Brodie et al., 2013), which is linked with frequent interaction with the focal object (Thakur, 2018). This study guides the marketing managers and retail practitioners significantly to incorporate SMMA to engage the potential customers, which will impact the sales intensity. As more and more e-retail businesses are moving toward social media usage to approach prospects as it is cost-efficient and effective for their attention. At present, people spend substantial time on social media activities, thus e-retailers may engage online shoppers.

**FIGURE 1:**
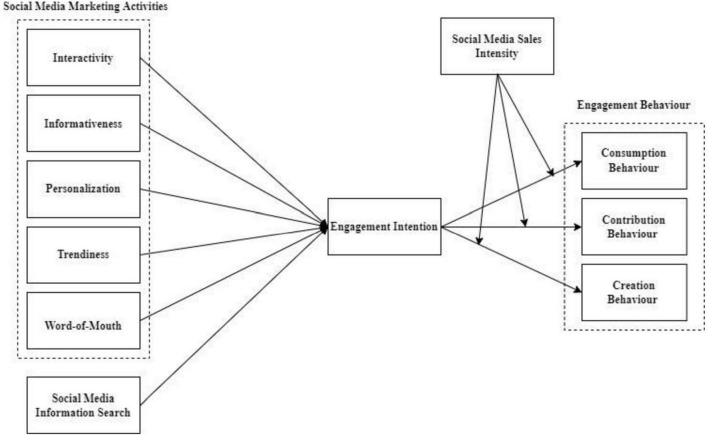
Conceptual model of the study.

This study unfolds a few shortcomings related to SMMA. First, a plethora of research has explored SMMA in a variety of contexts (Kautish and Sharma, 2019; Khare and Kautish, 2020). There is a severe dearth of studies with mediating and moderation relationship of SMMA (Kautish et al., 2021a). SMMA needs to be studied in the context of online shopping B2C e-commerce websites (e.g., Facebook, Instagram, and WhatsApp), which are gaining popularity in today’s emerging market (Kautish et al., 2021d). Second, SMMA are studied with reference to stimulus-organism-response (SOR) theory to investigate the relationship between SMMA and engagement (refer to Figure 1). Previously, SMMA was studied using the gratification theory (McGuire, 1974), which states that individuals’ motivation for media utilisation is to satisfy their perceived needs and desires. Third, limited attention is paid in practice to SMMA (Charoensukmongkol and Sasatanun, 2017; Cao et al., 2021), SMIS, and engagement intention corresponds with their significance to practitioners. Fourth, this study examined the moderating effect of social media sales intensity on the relationship between engagement intention and engagement behaviour. This study is based on the exhaustive review of previous studies conducted in the field of online shopping B2C e-commerce websites (Akram et al., 2018; Abumalloh et al., 2020). As technology companies, such as Amazon, are adapting to their customer’s specifications to explain the SMMA phenomenon (Kautish et al., 2021c). Cao et al. (2021) proposed customers engagement such as consumption, contribution, creation, and this facet was adapted in this study with SMMA (Kautish et al., 2022). This study attempts the moderating effects of social media sale intensity in the relationship between engagement intention and engagement behaviour. Additionally, Li et al. (2021) suggested that firms should use SMMA to engage online shoppers. Based on the previous recommendation and identified research gap, this study model is developed.

## Related Theory, Literature Review, and Research Hypotheses

### Stimulus-Organism-Response

Mehrabian and Russell (1974) originated the SOR model in 1974, and it stated that the environment as a stimulus (S) creates a set of signs of inner states, i.e., cognitive and affective reactions, and exhibits certain behaviours. In this study, perceived SMMA were used as stimuli. SOR stated that stimulus is an environment that consists of an internal evaluation of someone (O) and produces a response (R). The aim of the SOR model was to explain behavioural outcomes (consequences) in terms of internal cognitive and affective outcomes (of organisms) in certain environments (stimuli) (Chan et al., 2017). In this situation, engagement intention is termed as an inner state of customers or organisms (O) and may be categorised as an emotional state that occurs as a response to the stimulus. The response is the outcome based on the internal evaluation (O). In this study, engagement behaviour is the response; it is commonly observed that online social media evokes consumers’ emotions and affects their behaviour (Manganari et al., 2009). Here, SMMA are termed as a stimulus. SMMA are defined as “a process by which companies create, communicate, and deliver online marketing offerings *via* social media platforms to build and maintain relationships with stakeholders that enhance value to stakeholders by facilitating interaction, sharing information, offering personalised purchase recommendations about existing and trending products and services” (Yadav and Rahman, 2018, p. 3884). The characteristics of an e-commerce environment in which customers interact are categorised as stimuli (Eroglu et al., 2003). Emotional and cognitive states include their experiences, assessments, and insights (Gao and Bai, 2014), and responses symbolise the behaviour. Following the review of the literature, we will focus our attention on the factors that are related to SMMA, such as engagement intention, social media sales intensity, and engagement behaviour.

### Hypotheses Development

#### Social Media Marketing Activities and Engagement Intention

Social media marketing is defined as “a broad category of advertising spending, including advertising using the social network, virtual worlds, user-generated product reviews, blogger endorsement, RSS feeds of content and social news sites, podcasts, games, and consumer-generated advertising” (Bilgin, 2018, p. 129). Social media marketing platforms are linked to specific target consumers and strategies (Wibowo et al., 2020). SMMA resulted in both positive and negative outcomes. As a positive outcome, it generates customer retention and increases purchase intention (Hanaysha, 2018).

Interactivity is defined as “the extent to which e-commerce’s social media facilitates customers to share content and views with the company and other customers” (Ibrahim et al., 2020, p. 558). It is basically customer-generated content (Daugherty et al., 2008) and provides a platform for customers to share helpful content and crucial ideas about the product or services (Godey et al., 2016; Kautish and Rai, 2019). Interactive messages give the impression that companies are listening and responding (Bozkurt et al., 2021). The interactivity of social media empowers consumers to engage with companies and make decisions (Loureiro et al., 2019). Interactive stimuli involve a two-way communication and enhance customer engagement (France et al., 2016). Thus, the following hypothesis is proposed:

**H1:** Interactivity is positively related to engagement intention.

Informativeness is defined as the accuracy, comprehensiveness, and utility of the information a customer receives on social media. On social media, customers engage in e-shopping when they perceive that information about the product or service is accurate and sufficient to make a decision (Yadav and Rahman, 2018). This experience facilitates a favourable attitude toward the site (Elliott and Speck, 2005). Various e-commerce websites are easily evaluated on social media, and customers make the best choice (Aladwani and Palvia, 2002). This information stimulates consumer exposure and attention to predict engagement (Shareef et al., 2019). Informativeness stimulates conversation and reinforcement. Informative brand messages drive valuable customer engagement (Ul Islam and Rahman, 2017). Therefore, the following hypothesis is proposed:

**H2:** Informativeness is positively linked with engagement intention.

Personalisation refers to how social media is customised according to customer preferences (Koay et al., 2020; Kautish et al., 2021b). Customers share more individual experiences with social media (Martin and Todorov, 2010). Excessive information is available to customers’ personalisation in social media, scanning the information, and providing decision quality and superior e-shopping experience (Tam and Ho, 2006). Thus, we postulate that:

**H3:** Personalisation is positively linked with engagement intention.

Trendiness is defined as a particular social media dimension where customers perceive offers with more trendy content (Ebrahim, 2020). Social media trendiness has four main motivations: knowledge, surveillance, pre-purchase information, and inspiration (Voorveld et al., 2018). According to Muntinga et al. (2011), surveillance refers to keeping people informed about their surroundings, knowledge about the company’s product or services from other customers’ awareness and proficiency; pre-purchase mentions as product or services review, rating the recommendation from other customers to make a well-informed purchase decision, and acquiring as company relevant information and attaining innovation ideas. This trendy SMMA dimension informed customers about the latest fashion and stimulus engagement and facilitated better decisions (Godey et al., 2016; Kautish and Rai, 2018; Djafarova and Trofimenko, 2019). This stimulates customers’ sense of uniqueness and style to increase engagement (Ajitha and Sivakumar, 2019). Hence, the following hypothesis is proposed:

**H4:** Trendiness is positively linked with engagement intention.

Word-of-mouth (WOM) is the process of sharing and recommending the value of the experience of products and services on social media (Barreto, 2014). Berger (2014) focussed on WOM for the ownership, use, or features of a product or its seller. At present, WOM is replaced with e-WOM, as previews related to products and services are available online. WOM increases the widespread use of e-commerce and marketing (Yadav and Rahman, 2018). WOM increases customer satisfaction and reduces perceived risk (Park and Kim, 2008). Therefore, the following hypothesis is proposed:

**H5:** WOM is positively linked with engagement intention.

#### Social Media Information Search and Engagement Intention

Highly motivated customers spend more time searching for information about the product or services they want to purchase (Hwang et al., 2020). Information search refers to exploring different sources before making a purchase decision (Lim and An, 2021). Engagement is defined as the emotional connection between customers and a company (Dessart et al., 2015). The key element of engagement is the exchange of information between customers, and today’s companies are getting the advantage of using social media to engage in this activity (Erat et al., 2006). Trainor et al. (2014) stated that there are positive relationships between social media and the financial and non-financial performances of an organisation. Companies targeting customers using social media generate an intensive sale through it. Companies are also facing tough competition with other companies selling similar products or services (Charoensukmongkol and Sasatanun, 2017). SMMA categorise outcomes in communication, information processing, sales, and social responses (Ashley and Tuten, 2015). Rodriguez et al. (2014) stated that social media increases the sales performance of an organisation. Therefore, the following hypothesis is proposed:

**H6:** SMIS is positively linked with engagement intention.

Engagement is defined as the psychological state of mind. Engagement behaviour is operationalised into three dimensions, namely, consumption, contribution, and creation. Consumption engagement behaviour refers to the media created by the user and the firm. It represents the minimum level of engagement and the users consume it passively. Consumption mainly occurs through reading (Shao, 2009). Bolton et al. (2013) stated that consumption covers playing, searching, and assuming that the user reads these contents during a search. Contributions refer to a higher level of engagement, including peer-to-content and peer-to-peer interactions on social media. Creation refers to a higher level of engagement and is generated by the consumer and displayed on social media (Schivinski et al., 2016). Keller (2016, p. 11) defined engagement behaviour as “how different customers may want different relationships with a brand.” There were three types of users in this category; first, followers who read the content of the social media website (Zhu and Chen, 2015); second, opportunists who retrieve marginal information, such as recipes; third, functionalists who focus on information (de Valck et al., 2009). Thus, the following hypotheses are proposed:

**H7:** Engagement intention positively links with consumption engagement behaviour.
**H8:** Engagement intention positively links with contribution engagement behaviour.
**H9:** Engagement intention positively links with creation engagement behaviour.

#### The Moderating Role of Social Media Sales Intensity

People engaged in SMMA may feel the importance of this in increasing firm sales. The advantage of using SMMA for companies is that they increase the sales volume of products and services as compared with physical stores (Charoensukmongkol and Sasatanun, 2017). To increase sales using social media, companies should target specific customers. If the company uses SMMA more effectively, it may engage in the behaviour. SMMA used in the best way helps the company achieve differentiation and acquire more sales compared with companies that do not use social media. Companies that use SMMA to boost sales and provide superior services to customers are considered the best (Ainin et al., 2015). Using social media to increase companies’ sales is more dominant in companies with high IT intensity than in those with low IT intensity (Ravichandran et al., 2005; Zhu and Chen, 2015; Mehmood and Hussain, 2017a,b; Mehmood et al., 2021a). When consumers rate a product or service online, it influences sales (Chevalier and Mayzlin, 2006). Social media is more effective in terms of sales than cost (Dinner et al., 2014). As social media targets more consumers and engages them through the options of sharing, liking, and commenting, they influence purchase decisions and sales (Atsmon et al., 2012). Companies use technology to engage customers and increase sales (Eisingerich et al., 2019).

Li et al. (2021) proposed that the use of social media by firms increases or stimulates sales. Järvinen and Taiminen (2016) suggested that SMMA should be integrated with sales departments to gain insight into how to engage customers and synergise their efforts. Utilising social media content to attract customers increases their engagement behaviour and boosts the sales of products or services (Malthouse et al., 2013). Various authors (Algharabat et al., 2018; Kapoor et al., 2018; Kaur et al., 2018; Dwivedi et al., 2021) have argued that firms use social media to engage with their customers by creating positive attitudes toward products or services, getting feedback, improving brand awareness, and increasing sales. Most companies use social media to connect with existing and new customers to create awareness about brands and images and to increase sales (Choi et al., 2016; Yadav and Rahman, 2016; Kunja and GVRK, 2018). Hence, it can be concluded that social media sales moderate the positive link between engagement intention and engagement behaviour. Thus, the following hypotheses are proposed:

**H10:** Social media sales intensity intensifies the predictive influence of engagement intention on contribution behaviour in such a way that as social media sales intensity increases; this association will become stronger.
**H11:** Social media sales intensity intensifies the predictive influence of engagement intention on consumption behaviour in such a way that as social media sales intensity increases; this association will become stronger.
**H12:** Social media sales intensity intensifies the predictive influence of engagement intention on creation behaviour in such a way that as social media sales intensity increases; this association will become stronger.

## Materials and Methods

### Data Collection

Data from Hootsuite (2021) reported 61.34 million internet users in Pakistan by the end of January 2021, with an increase of approximately 21% from 2020 to 2021. Annually, a 24.3% (9 million) change was observed in social media users. It was reported that 40 million audience reports on Facebook, 11 million on Instagram, and 2.1 million on Twitter. The importance of SMMA can be judged by highlighting the statistics of US$25.85 million spent on digital advertisements in 2020. The data for this study were gathered purposively from Pakistan. To achieve the objectives of this study, the unit of analysis was regular social media users, such as Facebook, Instagram, and Twitter, who purchase online products based on the reviews or ratings *via* SMMA, following the previous studies (Yadav and Rahman, 2018; Feng et al., 2021). As many potential customers check the reviews and ratings of the previous buyers. For this study, all consumer products are taken into account like cosmetics, shoes, bags, clothes, kitchen items, and watches. There is a clear advantage of the SMMA as people can easily evaluate the available options through ratings and reviews given by the previous customers on e-commerce websites. Before the commencement of the formal investigation, respondents were assured that their involvement would be completely confidential, and they would get PKR 300 as a reward for their participation.

In this study, data were collected at two-time intervals using the time-lagged approach. Two waves of data collection were undertaken at 2-month intervals to reduce common method bias (Podsakoff et al., 2003; Begum et al., 2020; Khan, 2021a,b; Khan et al., 2021; Ullah et al., 2021; Yu et al., 2021). A total of 800 people were surveyed in the first wave (T_1_) of the study. A total of 660 questionnaires were completed and returned with a response rate of 82.5%. Participants were requested to report their perception of SMMA, SMIS, engagement intention, and social media sales intensity and provide their demographic information. A total of 660 respondents were asked to report their engagement behaviour in a second-wave survey (T_2_), which took place 2 months later. The number of usable responses returned was 396 with a response rate of 60%. The study sample comprised 206 (52.0%) men and 190 (48.5%) women. The respondents’ average age was 27 years, 22.7% had a bachelor’s degree, and 51.8% had a master’s degree. The average income of the respondents was approximately PKR 500,000.

### Measures

For the research constructs and demographic variables, the questionnaires employed scales adapted from previous studies. The Likert scale ranged from 1 to 5, with 1 denoting “strongly disagree” and 5 denoting “strongly agree.” A back-translation approach was used to convert the original English questionnaire into Urdu (Brislin, 1980; Mehmood and Li, 2018; Khan et al., 2020; Khan, 2021c; Khan and Khan, 2021). To measure perceived SMMA, interactivity (α = 0.957); sample item of this scale was “The social media allows me to share and update the existing content,” informativeness (α = 0.901); sample item of this scale was “The social media offers accurate information on products,” personalisation (α = 0.923); sample item of this scale was “The social media facilitates personalised information search,” trendiness (α = 0.942); sample item of this scale was “Contents visible on the social media is the latest trend,” and word-of-mouth (α = 0.960); sample item of this scale was “I would recommend my friends to visit the social media,” a 3-item scale for each was used, drawn from Yadav and Rahman (2018). To measure SMIS (α = 0.919), we used a 3-item scale developed by Javed et al. (2020). The sample item of this scale was “I think social media sites help to search credible, and reliable information for making rational decisions.” We used a four-item scale developed by Taylor and Todd (1995) to measure engagement intention (α = 0.922), and later applied by Cao et al. (2021); sample item of this scale was “I will not hesitate to engage with social media marketing for information or communication with people.” An eight-item scale was used to measure social media sales intensity (α = 0.954), adapted from Charoensukmongkol and Sasatanun (2017). To assess engagement behaviour, consumption behaviour (α = 0.826); sample item was “I followed a poster/picture/graphics related to the product,” contribution behaviour (α = 0.922); sample item was “I recommended online products,” and creation behaviour (α = 0.939); sample item was “I initiated a discussion related to the online product,” 4-item scale for each was adapted from Schivinski et al. (2016).

## Results

### Descriptive Statistics and Confirmatory Factor Analysis

Before analysing the data, the assumptions for both univariate and multivariate were checked. All studied variables were examined for missing values, outliers, homoscedasticity, normality, and multicollinearity. The dataset’s skewness and kurtosis values show that it is normally distributed. It was determined that the linearity and multicollinearity analysis scales are between −1.89 and +0.392, respectively. Multicollinearities were examined in this study by VIF (i.e., variance inflation factor) performed by SPSS 24.0. All values of the VIFs are below the threshold value of 10 (Hair et al., 2010), hence there is no multicollinearity concern in our study. Therefore, these results established that the dataset is appropriate for the regression analysis. Table 1 shows the internal reliabilities of the predictors and outcome variables including means and standard deviations. Correlations between the study’s variables are compatible and give preliminary evidence for hypothesis testing. We conducted a confirmatory factor analysis (CFA) by utilising AMOS 24.0 of the studied variables in accordance with Anderson and Gerbing’s (1988). To examine convergent and discriminant validity, Hu and Bentler’s (1999) cut-off criteria (i.e., χ^2^/*df* less than 2, CFI greater than 0.90, and RMSEA less than 0.07) were utilised. To establish the data’s validity, we used CFA on individual-level data with multiple item variables. The findings of the CFA analysis are shown in Table 2. When compared to other models, the baseline model test results revealed that the 11 factor had a satisfactory match with the data (χ^2^/*df* = 1,116.324/685 = 1.630; CFI = 0.969; TLI = 0.964; and RMSEA = 0.040). The factor loadings (λ = with cut-off criterion more than 0.60 and *p* < 0.001) were all greater than 0.610, as shown in Table 3, and all items examined demonstrated significant loadings on their related factors. We also evaluated AVE (cut-off criterion >0.50) and CR (cut-off criterion >0.80), both of which confirmed convergent validity (Table 3). As a result, the suggested model was found appropriate for hypothesis testing.

**TABLE 1:** Descriptive statistics, reliabilities, and correlation matrix.

	Mean	SD	1	2	3	4	5	6	7	8	9	10	11
1. Interactivity	3.303	1.443	(0.957)										
2. Informativeness	3.008	1.400	0.338**	(0.901)									
3. Personalisation	2.888	1.369	0.024	0.040	(0.923)								
4. Trendiness	2.984	1.462	0.022	0.016	0.019	(0.942)							
5. Word-of-mouth	3.388	1.406	0.039	0.004	0.169**	0.025	(0.960)						
6. Social media information search	2.893	1.398	0.354**	0.128*	0.002	0.047	0.013	(0.919)					
7. Engagement intention	3.701	1.290	0.101*	0.104*	0.112*	0.121*	0.103*	0.357**	(0.922)				
8. Social media sales intensity	3.074	1.517	0.167**	0.014	0.012	0.098	0.003	0.435**	0.269**	(0.954)			
9. Consumption behaviour	4.060	0.710	0.078	0.079	0.010	0.055	0.059	0.129*	0.220**	0.337**	(0.826)		
10. Contribution behaviour	2.715	1.127	0.158**	0.084	0.102*	0.121*	0.033	0.063	0.141**	0.102*	0.089	(0.922)	
11. Creation behaviour	3.669	1.111	0.071	0.055	0.004	0.022	0.032	0.047	0.108*	0.212**	0.338**	0.027	(0.939)

**p < 0.01, *p < 0.05; N = 396; Cronbach’s α values are displayed along diagonal.

**TABLE 2:** Confirmatory factor analysis.

Model	χ^2^	*df*	χ*^2^/df*	Δχ^2^ (Δ*df*)	TLI	CFI	RMSEA
Eleven-factor model: baseline model	1,116.324	685	1.630		0.964	0.969	0.040
Ten-factor model: combining SMIS, CTB, and CRB	4,094.170	738	5.547	2,977.846 (53)	0.743	0.756	0.107
Nine-factor model: combining SMSI, COB, CTB, and CRB	4,659.943	738	6.314	3,543.619 (53)	0.700	0.715	0.116
Eight-factor model: combining INT, INF, PER, TRE, WOM, and SMIS	6,250.668	738	8.469	5,134.344 (53)	0.578	0.600	0.137
Seven-factor model: combining INT, INF, PER, TRE, WOM, and EI	6,705.940	738	9.086	5,589.616 (53)	0.543	0.567	0.143
Six-factor model: combining EI, SMSI, COB, CTB, and CRB	6,727.182	738	9.115	5,610.858 (53)	0.542	0.565	0.143
Five-factor model: combining PER, TRE, WOM, SMSI, EI, and COB	8,064.542	738	10.927	6,948.2158 (53)	0.439	0.468	0.158
Four-factor model: combining TRE, WOM, SMSI, EI, COB, and CTB	8,102.399	739	10.964	6,986.075 (54)	0.436	0.465	0.159
Three-factor model: combining WOM, SMSI, EI, COB, CTB, and CRB	8,218.518	739	11.121	7,102.194 (54)	0.427	0.457	0.160
Two-factor model: combining INF, PER, TRE, WOM, SMSI, and EI	8,450.006	739	11.434	4,333.682 (54)	0.410	0.440	0.162
One-factor model: combining all into one factor	12,809.636	741	17.286	11,693.312 (56)	0.176	0.123	0.203

*INT, interactivity; INF, informativeness; PER, personalisation; TRE, trendiness; WOM, word-of-mouth; SMIS, social media information search; EI, engagement intention; SMSI, social media sales intensity; COB, consumption behaviour; CTB, contribution behaviour; CRB, creation behaviour; TLI, Tucker-Lewis’s index; CFI, comparative fit index; RMSEA, root-mean-square error of approximation.*

**TABLE 3:** Variable’s reliabilities and convergent validity.

Variables	Items code	λ	CR	AVE
Interactivity (INT), (Time-1)	INT1–INT3	0.917–0.951	0.957	0.882
Informativeness (INF), (Time-1)	INF1–INF3	0.810–0.924	0.902	0.755
Personalisation (PER), (Time-1)	PER1–PER3	0.848–0.930	0.924	0.803
Trendiness (TRE), (Time-1)	TRE1–TRE3	0.880–0.946	0.943	0.846
Word-of-mouth (WOM), (Time-1)	WOM1–WOM3	0.942–0.949	0.960	0.889
Social media information search (SMIS), (Time-1)	SMIS1–SMIS3	0.854–0.909	0.919	0.791
Engagement intention (EI), (Time-1)	EI1–EI4	0.825–0.885	0.923	0.750
Social media sales intensity (SMSI), (Time-1)	SMSI1–SMSI8	0.839–0.930	0.954	0.777
Consumption behaviour (COB), (Time-2)	COB1–COB4	0.610–0.851	0.830	0.553
Contribution behaviour (CTB), (Time-2)	CTB1–CTB4	0.835–0.913	0.922	0.748
Creation behaviour (CRB), (Time-2)	CRB1–CRB4	0.857–0.922	0.939	0.795

*All factor loadings are significant at (p < 0.001), N = 396; λ, factor loadings. AVE, average variance extracted; CR, composite reliabilities.*

### Structural Model and Hypothesis Testing

**FIGURE 2:**
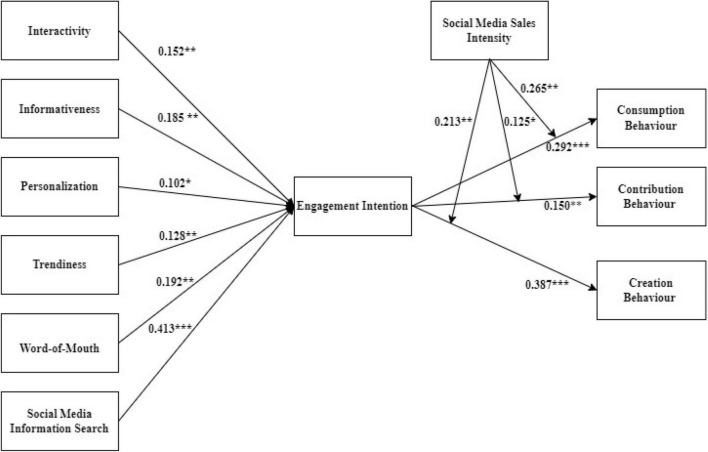
Path analysis results; ***p < 0.001, **p < 0.01, *p < 0.05.

We employed the structural equation model (SEM) technique to analyse the conceptual model, which has been extensively used in previous studies (Mehmood et al., 2019; Shang et al., 2021; Albloushi et al., 2022). The suggested model has a good fit, according to the fit indices (χ^2^/*df* = 877.475/158 = 1.694, CFI = 0.968, TLI = 0.965, and RMSEA = 0.042). As exhibited in Figure 2, interactivity is positively related to engagement intention (β = 0.152, *t* = 3.140, *p* < 0.01), which confirms H1. According to the path analysis, informativeness significantly influences engagement intention (β = 0.185, *t* = 4.541, *p* < 0.01), which confirms H2. H3 proposed that personalisation significantly influences engagement intentions. The path analysis revealed that personalisation significantly influences engagement intention (β = 0.102, *t* = 2.130, *p* < 0.05), which confirms H3. H4 proposed that trendiness significantly influences engagement intentions. The path analysis revealed that trendiness significantly influences engagement intention (β = 0.128, *t* = 2.542, *p* < 0.05), which confirms H4. H5 proposed that WOM significantly influences engagement intentions. The path analysis reveals that WOM significantly influences engagement intention (β = 0.192, *t* = 2.943, *p* < 0.05), which confirms H5. H6 proposed that SMISs significantly influence engagement intentions. The path analysis reveals that SMIS significantly influences engagement intention (β = 0.413, *t* = 7.639, *p* < 0.0001), which confirms H6.

Hypothesis 7 proposed that engagement intention significantly influences consumption behaviour. According to the path analysis (Figure 2), engagement intention significantly influences consumption behaviour (β = 0.292, *t* = 4.427, *p* < 0.0001), which confirms H7. As exhibited in Figure 2, engagement intention significantly influences contribution behaviour (β = 0.150, *t* = 2.798, *p* < 0.01), which confirms H8. As shown in Figure 2, engagement intention significantly influences creation behaviour (β = 0.387, *t* = 6.836, *p* < 0.0001), confirming H9.

**FIGURE 3:**
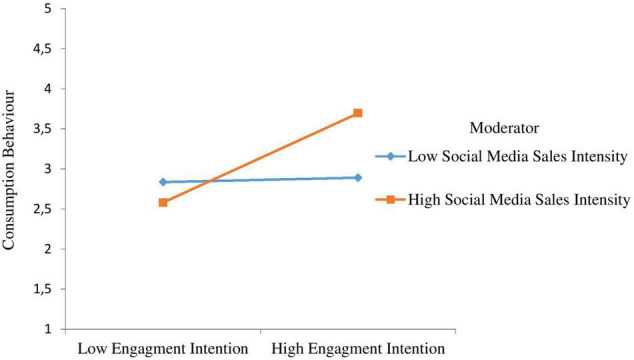
Interactive effects of engagement intention and social media sales intensity on consumption behaviour.

Hypothesis 10 anticipated that at higher levels of social media sales intensity, the positive link between engagement intention and consumers’ consumption behaviour will be stronger. As shown in Figure 2, the interaction between engagement intention and social media sales intensity predicted consumers’ consumption behaviour significantly (β = 0.265, *t* = 4.222, *p* < 0.01), which supports H10. Furthermore, we plotted the interaction in accordance with Aiken and West’s (1991) and Figure 3 indicates that higher levels of social media sales intensity, the link between engagement intention and consumers’ consumption behaviour grows stronger, confirming H10.

**FIGURE 4:**
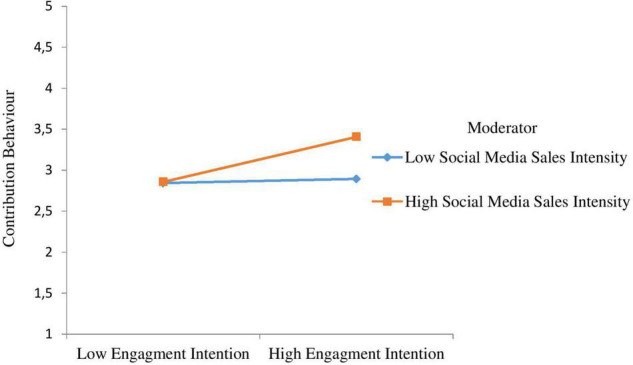
Interactive effects of engagement intention and social media sales intensity on contribution behaviour.

Hypothesis 11 anticipated that at higher levels of social media sales intensity, the positive association between engagement intention and consumers’ contribution behaviour will be stronger. As shown in Figure 2, the interaction between engagement intention and social media sales intensity predicted consumers’ contribution behaviour significantly (β = 0.125, *t* = 2.280, *p* < 0.05), which supports H11. We also plotted the interaction in accordance with Aiken and West’s (1991) and Figure 4 indicates that higher levels of social media sales intensity, the association among engagement intention, and consumers’ contribution behaviour grows stronger, confirming H11.

**FIGURE 5:**
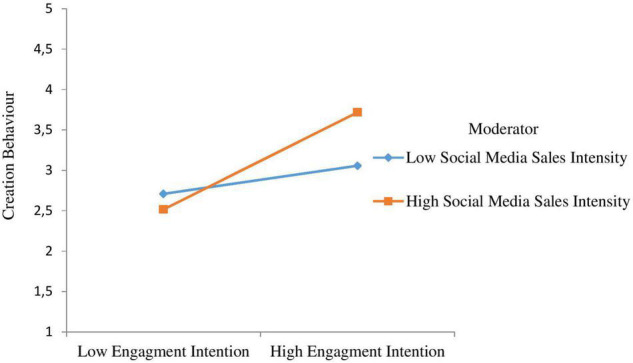
Interactive effects of engagement intention and social media sales intensity on creation.

Hypothesis 12 anticipated that at higher levels of social media sales intensity, the positive association between engagement intention and consumers’ creation behaviour will be stronger. As shown in Figure 2, the interaction between engagement intention and social media sales intensity predicted consumers’ creation behaviour significantly (β = 0.213, *t* = 3.921, *p* < 0.01), which supports H11. We also plotted the interaction in accordance with Aiken and West’s (1991) and Figure 5 indicates that higher levels of social media sales intensity, the association between engagement intention and consumers’ creation behaviour grows stronger, confirming H12.

## Discussion and Conclusion

Based on the SOR theory, this research unfolds the mechanism through which SMMA affects engagement behaviour. This study’s findings confirm the hypotheses related to the links between SMMA, SMIS, engagement intention, engagement behaviour, and social media sales intensity. According to the study findings of the time-lagged design, it is found that SMMA are positively related to engagement intention; engagement behaviour is positively related to engagement behaviour. Moreover, social media sales intensity strengthens the link between engagement intention and engagement behaviour.

This research investigates SMMA and SMIS with the moderated role of social media sales intensity among engagement intention and engagement behaviour. Engagement intention is a significant predictor of engagement behaviour, and social media sales intensity is significantly related to engagement behaviour in all three dimensions, i.e., consumption, contribution, and creation. The study found a significant positive contribution of all dimensions to engagement intention. This study applied the SOR model to understand SMMA, social media search information, engagement behaviour with engagement intention, and social media sales intensity as a moderated effect. The findings show that stimuli (SMMA and SMIS) cause an organism (engagement intention), in turn, create a response (engagement behaviour).

The results suggest that SMMA is a significant predictor of engagement intention, and this finding is consistent with that (Syrdal and Briggs, 2018). The study proposed that engagement is considered a state of mind and is treated separately from liking and sharing content. The results of the study also suggest that SMISs significantly predict engagement intention. This result is consistent with Jacobsen and Munar’s (2012) study in which it was suggested that SMIS matters, but it does not predict the actual online shopping; it is just the intention to search for information among various alternatives. Engaged behaviour demonstrates that the companies involved in engaged opportunities give more importance to customers and respond to their needs (Heaphy and Dutton, 2008).

### Implications of the Study

#### Implications for Research

First, SMMA and engagement behaviour knowledge were extended by applying the SOR model. Second, engagement behaviour in terms of consumption, contribution, and creation association was examined with engagement intention. Third, the moderated effect of social media sales intensity was examined between engagement intention and engagement behaviour. Fourth, engagement intention and behaviour are most often checked in the fashion industry, but this study examined its effects on online shopping websites.

This study explicitly examined the role of environmental stimuli (SMMA and SMIS) in the development of organismic reactions (engagement intention), which shapes engagement behaviour. Using the SOR theory to explain the linkage between current research variables was also a contribution. The findings of this study provide strong evidence for SOR in the context of engagement behaviour. The application of SOR in such a context is rare in the literature, despite SOR being used in several other contexts such as retailing, the hotel industry, and organisational behaviour (Kandampully et al., 2018; Mehmood et al., 2020; Nunkoo et al., 2020). This study has several practical implications. Regulatory institutions provide insight into the mechanisms of SMMA and engagement behaviour. To achieve the effectiveness of the SMMA, careful selection of media will be given equal importance, which is appropriate for creating trendiness, personalisation, interactivity, informativeness, and WOM. As companies try to maintain and achieve maximum engagement, media selection will be of valuable importance. Second, SMMA plays a significant role in creating engagement intentions. Careful selection of the media should not be neglected. SMIS significantly influences engagement intention, which gathers and updates the relevant information and is available to all customers. Companies use this promotion technique, such as SMMA, to gain customer attention and maximise sales.

#### Implications for Practitioners

This study highlights the significance of marketers in giving attention to SMMA, engagement intention, and engagement behaviour. First, marketers should unfold the benefits of SMMA in the current scenario, where it becomes a necessity for all customers. This has become the most interactive, accessible, and appropriate medium for creating eWOM. As highlighted by Chan et al. (2014) and Hollebeek et al. (2016), it generates customer cognition, activation, and affection. Few researchers have recommended that companies engage customers by rewarding them to share their experiences and information on the SMMA (Wang et al., 2012; Sijoria et al., 2018). Moreover, businesses should invest in more personalised predictive recommendation engines for their social networking sites. As a result, such insightful recommendations will help to make consumers feel valued, resulting in a more favourable effective experience. An effective social media marketing strategy encourages customers to share product information of their own will through different activities like interaction and personalisation. Marketing managers may increase trendiness by routinely updating their social media online shopping sites to reflect the most recent news and offerings, so attracting customers’ attention and favourable emotions to the social media forums (Cheung et al., 2020).

Based on these findings, it is predicted that customers use SMMA extensively and are highly influenced by social media in the engagement process. Companies involved in SMMA can enhance sales intensity if they properly utilise the channels. SMMA and search information are becoming popular for evaluating products and services. SMMA is becoming an effective tool, as more customers are engaged with the company by utilising this resource. Companies should develop marketing strategies related to SMMA to increase consumption, contribution, and creation. Customers who use social media to check the posts, pictures, reviews, and ratings of a specific product or service increase sales, so it is recommended that those companies update and inform customers regularly to build relationships with customers through online communities. Companies must develop blogs, product review websites, or fan clubs to increase their image excellence *via* WOM.

E-commerce firms are putting efforts to increase online engagement behaviours, but they are unclear which medium provides specific kinds of engagement. Companies should devote their efforts to understanding the SMMA’s effect on engagement behaviour. Thus, personalisation, informativeness, and WOM, the dimensions of which increase maximum engagement, should be identified. Engagement behaviour is the desired outcome of a company, so managers should not underrate SMMA. Managers should devote considerable time to analysing which SMMA evokes the maximum engagement behaviour. Most companies are devoting time to creating platforms for customers on social media, but there is still a need to specify these efforts. This research also provides managers with the direction to measure engagement behaviour with the firm’s offering and activities on SMMA. Managers should focus on a blend of information exchange media and stores to promote engagement behaviour at high levels.

### Limitations

Time-lagged data were used in this study, which typically controls the common method bias (Podsakoff et al., 2012; Li and Mehmood, 2019; Mehmood et al., 2021a,b; Khan et al., 2022); hence, common source and common method biases were not major problems. Despite these advances, there are a few limitations of our study. First, the study’s data are from a single industry type in Pakistan and cannot be generalised to other sectors or national contexts. Second, this study included only Pakistani participants. The findings may be applicable to consumers in other collectivist nations, although this generalisation requires validation. Thus, future cross-cultural studies (Li et al., 2017, 2019; Jabeen et al., 2020; Mehmood et al., 2020, 2022; Alkatheeri et al., 2021; Mehrajunnisa et al., 2021; Müller et al., 2021) on Western and Eastern cultures might be interesting.

## Data Availability Statement

The raw data supporting the conclusions of this article will be made available by the authors, without undue reservation.

## Author Contributions

YS and KM have an equal contribution, conceived the idea and helped in writing the introduction and literature review. HR and YI collected the data. AX and RS wrote the discussion, conclusion, and implications of the study. YW helped in writing the literature review. All authors have read and approved the final manuscript.

## Author Disclaimer

The opinions and arguments expressed are those of the authors and do not represent views of the China Postdoctoral Science Foundation, National Natural Science Foundation of China, and Zhejiang Social Science Project.

## Conflict of Interest

The authors declare that the research was conducted in the absence of any commercial or financial relationships that could be construed as a potential conflict of interest.

## Publisher’s Note

All claims expressed in this article are solely those of the authors and do not necessarily represent those of their affiliated organizations, or those of the publisher, the editors and the reviewers. Any product that may be evaluated in this article, or claim that may be made by its manufacturer, is not guaranteed or endorsed by the publisher.
